# Assessing risk of bias: a proposal for a unified framework for observational studies and randomized trials

**DOI:** 10.1186/s12874-020-01115-7

**Published:** 2020-09-23

**Authors:** Hendrika J. Luijendijk, Matthew J. Page, Huibert Burger, Xander Koolman

**Affiliations:** 1 University of Groningen, University Medical Center Groningen, Department ofGeneral Practice and Elderly Care Medicine, Groningen, The Netherlands; 2 School of Public Health and Preventive Medicine, Monash University, Melbourne, Victoria Australia; 3 Department of Health Sciences, Vrije Universiteit Amsterdam, Amsterdam, The Netherlands

**Keywords:** Critical appraisal, Risk of bias, Validity, Randomized trial, Cohort study, Review

## Abstract

**Background:**

Evidence based medicine aims to integrate scientific evidence, clinical experience, and patient values and preferences. Individual health care professionals need to appraise the evidence from randomized trials and observational studies when guidelines are not yet available. To date, tools for assessment of bias and terminologies for bias are specific for each study design. Moreover, most tools appeal only to methodological knowledge to detect bias, not to subject matter knowledge, i.e. in-depth medical knowledge about a topic. We propose a unified framework that enables the coherent assessment of bias across designs.

**Methods:**

Epidemiologists traditionally distinguish between three types of bias in observational studies: confounding, information bias, and selection bias. These biases result from a common cause, systematic error in the measurement or common effect of the intervention and outcome respectively. We applied this conceptual framework to randomized trials and show how it can be used to identify bias. The three sources of bias were illustrated with graphs that visually represent researchers’ assumptions about the relationships between the investigated variables (causal diagrams).

**Results:**

Critical appraisal of evidence started with the definition of the research question in terms of the population of interest, the compared interventions and the main outcome. Next, we used causal diagrams to illustrate how each source of bias can lead to over- or underestimated treatment effects. Then, we discussed how randomization, blinded outcome measurement and intention-to-treat analysis minimize bias in trials. Finally, we identified study aspects that can only be appraised with subject matter knowledge, irrespective of study design.

**Conclusions:**

The unified framework encompassed the three main sources of bias for the effect of an assigned intervention on an outcome. It facilitated the integration of methodological and subject matter knowledge in the assessment of bias. We hope that graphical diagrams will help clarify debate among professionals by reducing misunderstandings based on different terminology for bias.

## Background

Evidence based medicine requires that individual physicians critically appraise scientific evidence. Guidelines may offer an overview of the evidence for many clinical situations, but may not be available or up to date. In addition, very old treatments, rare diseases and distinct patient groups are seldom covered in guidelines [1, 2]. In such cases, physicians will need to appraise the quality of relevant studies and interpret the results accordingly.

Nowadays, medical schools typically provide courses in the critical appraisal of research findings [3]. Critical appraisal starts with the definition of the clinical question in terms of the population of interest, the compared interventions and the main outcomes. Next, clinical relevance, reliability and validity of the study results need to be assessed. A reported effect of the intervention on the outcome is valid if it accurately reflects the real effect in the population of interest. If the effect was established with systematic error it is said to be biased. Risk of bias tools have been developed to help reviewers appraise studies in systematic reviews. Examples are the Jadad-score, Cochrane risk of bias tool, and the Mixed Methods Appraisal Tool [4–6].

However, the taxonomy of bias and terminology that is used differs across study designs (ref Schwartz). Different types of bias are identified, and even if they are structurally identical, different terms have been used to describe them. The lack of a straightforward and consistent framework for bias assessment across designs complicates bias assessment for health care professionals, and leads to confusion and unresolved semantic discussions. This is probably why few physicians assess bias thoroughly as part of their critical appraisals of studies.

In addition, use of subject matter knowledge is common in the assessment of bias in observational studies, but far less so in that of randomized trials [7, 8]. Subject matter knowledge refers to the facts, concepts, theories, and principles which are specific to a certain medical topic, e.g. cardiovascular medicine. For example, adjustment for baseline characteristics that are unequally distributed between treatment groups may be required if these variables are thought to be predictive of the outcome on the basis of subject matter knowledge (CONSORT) [7, 8]. It is commonly recommended to assess baseline differences in an observational study, but seldom in a randomized trial [9]. For most trial assessment tools, the focus is on checking the methodological aspects of design and execution, such as randomization procedures. Less attention is paid to understanding how the conduct of a trial in conjunction with the clinical context influenced the study findings. Thus, subject matter knowledge is indispensable for the assessment of bias in trial results too.

We propose a unified and simple framework to facilitate bias assessment for health care professionals, which is applicable to observational and experimental designs. It builds on an understanding of how bias originates and may be avoided. This knowledge then enables health professionals to use their subject matter knowledge and improve the appraisal of the evidence. In addition, students and clinicians make use of ‘pre-digested’ evidence more and more. The framework could also help people who pre-digest and summarize the evidence to perform a critical appraisal of the original evidence.

The framework has been accepted in observational epidemiology and underlines the prevailing taxonomy for bias. The identified sources of bias are not design dependent, so our goal was to show how the framework could be used to evaluate bias in trials, and teach bias assessment. As the framework stems from the literature about causal inference, i.e. the process of ascertaining that an effect is causal and not due to bias, this paper may also be regarded as an introduction to that literature [10].

## Methods

Epidemiological textbooks typically distinguish three sources of bias (described in more detail in the Results section) [11, 12]. First, the exposure and outcome have a cause in common. This *common cause* is called a confounder in epidemiology. If it is not adjusted for, confounding bias occurs. Second, there is *systematic measurement error* when (1) the exposure status influences the measurement of the outcome, (2) the outcome influences the measurement of the exposure, or (3) a third factor influences the measurement of both exposure and outcome. Such a measurement error, or (non-)differential misclassification, leads to information bias, also known as observation bias or measurement bias. Third, the exposure and the outcome both determine whether eligible patients participate in a study and whether all participants have been included in the analyses, e.g. a treatment and an adverse effect could have drop-out in common. In other words, exposure and outcome have *a common effect*. The selective drop-out of patients can result in selection bias.

For each source of bias, a causal diagram can be used to illustrate its mechanism. A causal diagram displays how the exposure of interest and the outcome of interest are associated as a result of the causal relationship of other variables with the exposure and outcome [10]. As such, the use of causal diagrams has facilitated identification of bias and adjustment for bias in observational studies [13].

We applied the framework for bias developed in observational studies to bias assessment in randomized trials. In the context of randomized trials, the ‘exposure’ is to be interpreted as the experimental intervention under study. The assessment started with the identification of the causal question and population of interest. Next, we discussed each source of bias, illustrated it with a causal diagram, and summarized which study designs and statistical techniques can be applied to minimize it. The sources of bias also indicated which study results should be assessed with subject matter knowledge. We have avoided the use of the terms confounding, information bias, and selection bias, because their meaning varies across epidemiological specialties (see online supplement) [12, 14].

## Results

### The causal question and population of interest

Risk of bias assessment begins with the identification of the causal question and population of interest (see Table 1 and eTable 1). What we usually want to know is: does intervention I affect outcome O in population P, and if so how strongly? Or in short: I → O in P?

**Table 1:**
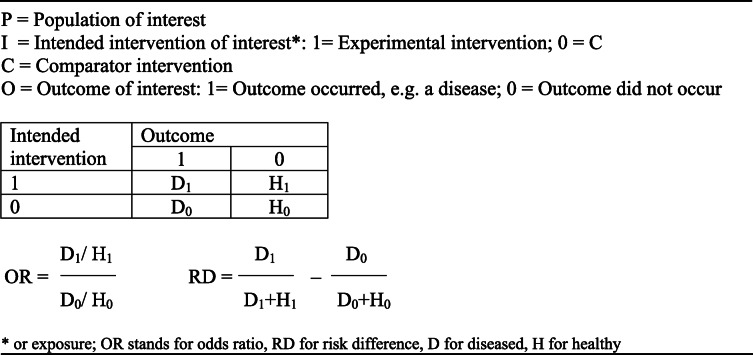
The causal question with 2 × 2 table, OR and RD

Population P is the target population to which the study results should apply. Usually, eligibility criteria determine which patients are included into a trial. These criteria as a rule do not coincide with the indications and contra-indications that health professionals take into account. Therefore, reviewers need to assess which eligibility criteria diminished the representativeness of the study population for the target population and how this could have affected the results.

Intervention I is a condition to which a patient can be exposed or not, e.g. one can be prescribed a drug that causes weight-loss or not; one cannot receive a certain weight or not [15]. Placebo is often used as the comparison intervention C to control for the natural course of the disease, be it improvement or deterioration, and the effect of unspecific treatment elements such as receiving attention. Pragmatic trials typically test the effectiveness of a new treatment versus standard treatment. In observational studies, on the other hand, the outcomes of a treatment are compared to no-use or another treatment. A reviewer needs to define a priori what control intervention is clinically relevant.

The effect of an intervention is defined in terms of clinically relevant, beneficial and harmful outcomes. The outcomes that trialists chose do not always reflect the outcomes that are important to patients, for instance a surrogate outcome such as serum LDL-cholesterol instead of clinical diseases such as myocardial infarction and stroke. The reviewer needs to determine a priori which outcomes reflect important health gains ànd losses. When the causal question has been determined and a study has been identified that addressed it, the next step is to assess how the methods could have biased the reported study results.

### Bias due to a common cause

The first possible source of bias is a factor - mostly a patient characteristic - that affected which intervention was assigned *and* influenced the risk of the outcome, independently. E.g. severity of disease could affect both the choice for a conventional antipsychotic drug and risk of death [16]. This is called a common cause [13]. This factor could explain a co-occurrence (association) between the intervention and outcome even if the intervention has no causal relationship with the outcome. Common causes can be measured or unmeasured.

**Fig. 1:**
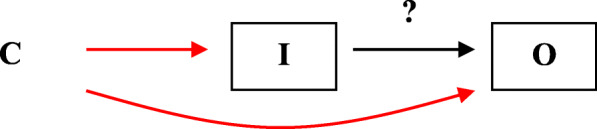
I stands for intended intervention, O for outcome, C for a common cause that differs between intervention groups. The arrow with question mark stands for the causal question (effect) of interest. Boxed nodes indicate variables in the analysis, i.e. C is not adjusted for

Figure 1 provides a causal diagram of bias due to a common cause. A causal diagram depicts the investigated effect of an intervention on an outcome (I → O), and other variables that influence the measured effect. In Fig. 1, the arrow with the question mark denotes the causal question (effect) of interest. The unmeasured patient characteristic C affects intervention I and outcome O, and it is not taken into account in the analysis (no box around the variable). The figure shows that even if there is no effect of I on O, an association between I and O will be found as a result of the ‘backdoor path’ via (backwards followed arrow from) I to C and C to O.

Bias due to known and unknown common causes can be avoided with randomization. Randomization, if performed correctly, ensures that chance determines which intervention a participant receives. Prognostic patient characteristics are expected to be equally distributed across treatment groups. Hence, assuming no other biases, differences in outcomes between groups can be attributed to differences in treatment. For randomization to be effective, the allocation sequence must be truly random and concealed from those persons responsible for allocating participants [17]. These prerequisites ensure that the persons involved in the allocation cannot foresee the next allocation and therefore cannot use knowledge of patient characteristics to (1) change the treatment or forestall recruitment until the desired intervention comes up (C → I), or (2) decide not to recruit the participant into the study at all (see eFigure 1). The reviewer must assess whether these prerequisites were met and whether modifications, such as stratified randomization or blocked randomization with small, fixed blocks, could have made the next allocation predictable [18].

A commonly held misconception is that blinding the persons who provide the intervention is an adequate way to conceal an allocation. Take for instance an invasive procedure such as surgery, where the person providing the intervention cannot be blinded. As long as the recruiter and allocators cannot foresee the next allocation, this unblinded design will not interfere with the randomization procedure. Conversely, active and placebo drug tablets with identical appearance and taste can blind those involved in giving the treatment. Yet, if the recruiters or allocators know the allocation sequence, the allocation can still be (foreseen and) tampered with.

It must be emphasized that even if designed and conducted perfectly, randomization cannot guarantee prognostic comparability of treatment groups. Therefore, the assessor must evaluate group differences in prognostic baseline characteristics [8, 19]. According to the CONSORT statement, a correctly reported trial will present the baseline characteristics for *all* randomized participants in each intervention group (http://www.consort-statement.org). Testing the statistical significance of baseline differences has little value for risk of bias assessment [20, 21]. Sample sizes are often too small for these tests to be informative at all, and differences that are statistically insignificant might still cause relevant bias. In large trials, statistically significant baseline differences might not always be large enough to be relevant. Therefore, reviewers must assess whether differences between groups at baseline could explain the variations in outcomes irrespective of statistical significance. For instance, in a large trial testing the long-term safety of a drug for diabetes mellitus, the majority of characteristics that predict cardiovascular disease and death were distributed in favor of the drug versus the placebo group. As the incomparability of groups was not adjusted for, an underestimated risk of all-cause mortality cannot be ruled out [22]. When reviewing a set of trials for systematic review though, systematic baseline differences across trials and the distribution of *p*-values could indicate failed randomization [23–26].

In trials and observational studies, restriction of the study population to one stratum of a known common cause could also be used to avoid bias. If avoidance of bias due to a known common cause cannot be prevented by design, this type of bias can be adjusted for in the analyses if the common cause is measured well. Commonly used approaches include multivariable regression and propensity scores. Subject matter knowledge is essential to decide which characteristics need to be adjusted for [13].

### Bias due to systematic measurement error

The second type of bias is caused by systematic error in the measurement of the intervention status or outcome. Intervention status refers to the study intervention that a participant receives, that is the active drug or comparison intervention. Systematic measurement error could be caused by (1) the intervention status influencing the measurement of the outcome, (2) the outcome influencing the measurement of the intervention status, (3) or a third factor that causes systematic error in measurement of both the intervention and the outcome status. The first type of measurement error is important for randomized trials. If the outcome assessor (e.g. patient, health care provider, researcher) is aware of the participant’s study group at some time during the trial, this could systematically influence assessments. E.g. an assessor could report or register a more favorable result if expectations of the new treatment are high, or a less favorable result if expectations are low. This bias is often referred to with the term detection bias.

**Fig. 2:**
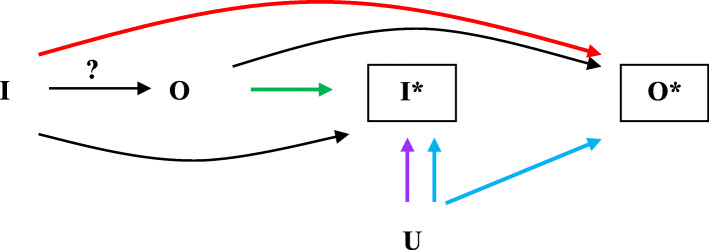
I stands for true intervention status, I* for measured intervention status, O for true outcome status, O* for measured outcome status, and U for a third (usually unmeasured) variable. The arrow with question mark stands for the causal question (effect) of interest. The red arrow signifies that Intervention I affects measured outcome O*, the green arrow that Outcome O affects measured intervention I*, the purple arrow that a third factor U affects measured intervention I*, and the blue arrow that a third factor U affects measured intervention I* and outcome O*. Boxed nodes indicate variables in the analysis

Figure 2 represents the three types of systematic measurement error, with I standing for true intervention, I* for intervention measured with error, O for true outcome, O* for outcome measured with error. The graph illustrates that even if there is no effect of I on O, an association between I and O will be found as a result of the path of arrows from I to O* and (backwards) O* to O.

The outcome can affect the measurement of the intervention (O → I*) only if the outcome has already occurred. A prospective design, whereby patients are recruited prior to the outcome, can be utilized to avoid this type of measurement error (eTable 1). To circumvent the intervention status influencing the outcome measurement (I → O*), outcome reporters and assessors need to be blinded to the intervention status. Reviewers should use subject matter knowledge to assess whether the method of blinding was (partially) effective. For instance, in spite of the identical appearance of active and placebo tablets, specific adverse events or the presence of the health professional providing the intervention could reveal which intervention was given [27]. Finally, a third -often unmeasured- factor could systematically affect the measurement of the intervention (U → I*), or the measurement of both treatment and outcome (U → I* and U → O*).

Measurement error may also be random, i.e. not systematically related to other variables. Random error in the intervention status will bias the estimated effect toward the null. This is often referred to as regression dilution. Random measurement error in the outcome does not result in bias. It will, however, lower the statistical power and increase the width of the confidence interval.

### Bias due to a common effect

The third type of bias occurs when both intervention and outcome determine whether certain eligible patients are not included in a study, or left out of the analysis [28]. This common effect, often referred to with the term selection, drop-out or attrition, can occur before or during a study. Selections based on intervention and outcome, whether before or after the start of a study, will reduce the validity of the study results to the target population.

A well-known example of bias due to drop-out occurs when trial participants discontinue the experimental treatment due to adverse effects. If disease deterioration also determines drop-out, an association between treatment and disease status at the end of the trial will be found, even if there is no real treatment effect. A lesser-known source of bias arises by de-selection of patients after a run-in period [29]. This period between screening and randomization is used to stop medications that are identical or similar to the experimental drug (wash-out), to administer placebo treatment in order to identify placebo responders or compliant patients, or to give the active treatment to identify intolerant patients. The selection of patients into the randomized phase of the study is based on their outcomes during the run-in period, such as an occurrence of, or a decrease in side-effects. Treatment response and side effects obtained in this selected population will not be similar to those in the population included at screening and may not represent the target population [30, 31]. The reviewer should therefore assess whether the results in the selected population can be generalized to the target population. A similar bias occurs when a cohort study is based on prevalent instead of first-time (incident) users [32, 33]. Drop-out during an observational study due to the effects of the treatment can introduce bias too: patients with a positive balance between beneficial and harmful reactions are probably overrepresented in the analyzed population.

**Fig. 3:**
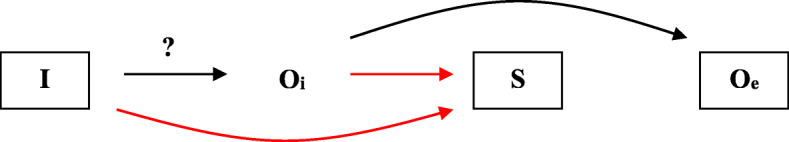
I stands for treatment status, O_i_ for intermediate outcome status, O_e_ for outcome status at endpoint, and S for a common effect (selection). The arrow with question mark stands for the causal question (effect) of interest. The box around S signifies that exclusion of patients based on treatment and outcome occurred as a result of the design or the analysis

Bias due to a common effect, or selection, is represented in Fig. 3. In the graph, intervention I and outcome O_i_ at time point i during follow-up lead to selection S. In other words, patients are selected out of the study. The effect of I on O was conditioned on S, which can lead to bias [34].

Bias due to selection (exclusion) can only be avoided if a study is based on first-time users and complete follow-up irrespective of treatment or outcome during follow-up (eTable 1). A valid trial design should not have exclusion criteria relating to effectiveness of prior (similar) interventions, nor exclude patients during run-in periods based on their response to active or placebo treatment during this period. In order to be informative for medical practice, a trial should include new users that are representative of patients in daily medical practice. For instance, in a trial about a drug for influenza, enrichment of the population with participants who were expected to show a favorable response, may have obscured the drug’s lack of effect in North-American adults [35]. This type of selection should be distinguished from excluding patients with certain contra-indications from participation (non-eligibility). These patients do not belong to the population of interest and therefore the effect of treatment in these patients is irrelevant. An observational study based on incident users avoids bias due to selection before the start of the study too.

To assess selection, a flow-chart needs to show drop-out before and after the start of the study. Reviewers should use subject matter knowledge to assess how drop-out could have affected the reported treatment effect. Preferably, reasons for and proportion of drop-out should be similar across comparison groups, although this certainly does not guarantee absence of bias [36]. In an intention-to-treat analysis, all participants are included in the intervention group to which they were allocated, irrespective of whether they actually received this intervention or completed the study. Modified ITT-analysis and per protocol-analysis exclude participants from the data-analysis [37]. As these are often non-completers, and completion frequently depends on (the lack of) efficacy or occurrence of side-effects (see flow-diagrams of trials), the selection is based on outcomes and likely to introduce bias.

### Combinations of biases

The three types of bias can co-occur. For example, baseline imbalance between study groups can affect selection based on treatment and outcome during follow-up. An example is given in Table 2. To address this, a reviewer needs to assess the risk of bias due to common causes as explained earlier.

**Table 2:**
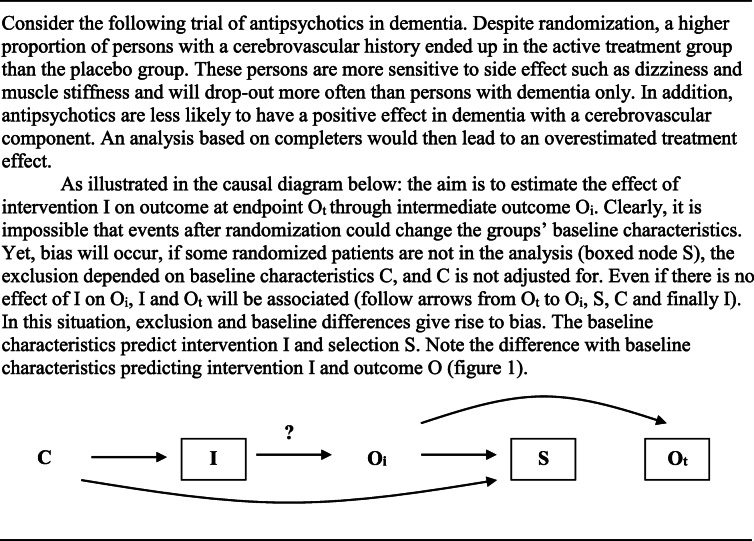
An example: when baseline differences and attrition are related

## Discussion

Evidence based medicine requires physicians and other health professionals to appraise the validity of scientific evidence. We have applied a framework which is popular for the assessment of bias in observational studies, to randomized trials. The framework identifies three sources of bias and these are independent of study design. After formulating the causal question, physicians can assess potential sources of bias using their methodological and subject matter knowledge. ETable 1 provides an overview of this approach. As such, our paper complements a previous publication that described the biases identified in the Cochrane tool for risk of bias with causal diagrams [38].

A clear advantage of the framework is its consistency and the use of terminology-free causal diagrams. In addition, it is robust to (future) modifications of conventional study design, such as run-in periods in trials, because it covers all potential sources of bias. Moreover, as the framework facilitates consideration of subject matter knowledge, bias assessment within and across study designs may gain more depth and consistency. The framework could therefore be useful for reviews covering both randomized trials and observational studies. A limitation of our approach is that it requires readers to learn the lexicon of causal diagrams.

We did not discuss protocol deviations in trials. In most observational studies and in some trials, the experimental and comparison intervention may not be static. Content and timing can change during follow-up, other treatments may be added, patients and health professionals may not comply well, or the treatment may be cancelled altogether. If such changes to the intervention are not permitted according to the protocol, they are called protocol deviations. We did not consider protocol deviations as a cause of bias in the effect of the *allocated* intervention I on outcome O, provided they are reflective of routine care [38]. Such deviations are part of and the result of the allocation (a so-called intermediate effect). Blinding of patients, caregivers, and attending health care professionals in trials can avoid some protocol deviations [17]. Yet, a properly blinded patient or health care professional might still initiate additional treatments, change or stop allocated treatment when the desired effects are not occurring. Therefore, trial articles usually report whether the *intended* experimental versus comparison intervention yields a treatment effect on average for a group of patients. Nevertheless, a detailed description and assessment of such protocol deviations, or intermediate effects, are important aspects of an appraisal. They might be responsible for the reported effect of the allocated treatment.

## Conclusion

A framework based on three sources of bias has supported the critical appraisal of observational studies. The three sources of bias are: a common cause of the intervention and outcome, a systematic error in the measurement of the intervention or outcome, and a common effect of the intervention and outcome. We applied the framework to randomized trials so that health professionals can use it to assess risk of bias of such studies. The unified framework may also be helpful for readers who aim to integrate evidence from both observational studies and randomized trials in a consistent assessment. Using the framework stimulates the interpretation of study results in relation to study design with subject matter knowledge.

## Supplementary information

**Additional file 1.**

## Data Availability

Not applicable.

## Abbreviation

CONSORT: CONsolidated Standards of Reporting Trials
