# Supplementary material for: Assessing risk of bias: a proposal for a unified framework for observational studies and randomized trials
Source: BMC Med Res Methodol. 2020 Sep 23;20:237. doi: 10.1186/s12874-020-01115-7 (PMC7510067; doi:10.1186/s12874-020-01115-7)
Supplement: Supplementary file 1 — Additional file 1. [file 12874_2020_1115_MOESM1_ESM.docx]

**Web attachment**

**eTable 1 Framework for the assessment of bias in trials and observational studies**

| **Domain** | **Methods to avoid bias** | **Results that suggest bias** | **Statistical adjustment for bias** |
| --- | --- | --- | --- |
| Causal question:  I → O in P? | Not applicable | Not applicable |  |
| Common cause C:  C → I and C → Y | **Randomization** (including a truly random sequence and allocation concealment to all persons involved in recruitment and allocation)  **Restriction** of study population to one stratum of a known C | **Baseline imbalance** (differences) between study groups in reported common causes  **Unmeasured and unreported** common causes | **E.g. stratification, multivariable regression**  **G-methods, Instrumental variables** |
| Measurement error due to unmeasured factor U:  O → I*,  I → O*,  U → I*, or  U → I* and U → O* | **Prospective design** to prevent the outcome from influencing measurement of intervention  **Blinding** **of outcome assessors** (investigator, health care professional, patient, caregiver) to intervention status  **‘Hard’ outcome** not sensitive to error | **Case-control study** (not nested in cohort study)  **Characteristics of the intervention that might unblind** (e.g. very specific side effects of the interventions)  **Reliability and validity of measurement instruments of soft outcomes** | -  -  - |
| Common effect S:  I → S and O → S | **Inclusions of new users**/ no exclusion relating to outcomes during prior use or run-in period (wash-out)  **Complete outcome assessment** **until end of study** **for all participants** included in the study irrespective of intervention status at the start or adherence during follow-up | -  **Differential drop-out** (numbers and reasons) | -  **ITT analysis with multiple imputation** **for missing data** (not LOCF) |

I stands for intervention, I* for measured intervention, O for outcome, O* for measured outcome.

**Terminology**

Different terms are used in epidemiology to refer to similar sources of bias. Etable2 provides an overview; it is not comprehensive but illustrates the problem. Baseline differences between comparison groups are also called baseline imbalance or prognostic risk differences. An ‘open label trial’ is a trial without blinding of treatment. Clinical studies with two or more comparison groups are sometimes called trials even if participants were not randomized.

**eTable 2 Terminology for bias in clinical and observational epidemiology**

| **Source of bias** | **Clinical epidemiology** | | **Observational epidemiology** | |
| --- | --- | --- | --- | --- |
|  | ***Terminology*** | ***Method to avoid this bias*** | ***Terminology*** | ***Method to minimise this bias*** |
| A common cause:  a factor that affects treatment and outcome is not adjusted for | Selection bias  Baseline imbalance  Bias arising from the randomization process | Randomization using a truly random sequence generation  and concealment of allocation^ | Confounding | Valid and reliable measurement and adjustment for baseline characteristic  Instrumental variable technique |
| Measurement error:  - treatment affects measurement of outcome;  - outcome affects the measurement of treatment;  -a third factor affects the measurement of treatment, or  -a third factor affects the measurement of treatment and outcome | Detection bias  Bias in measurement of outcome  -  Residual confounding  - | Blinding of treatment to patients and outcome assessors  -  -  - | Information bias (differential, independent)  Information bias  (differential, independent)  Information bias  (non-differential, independent)  Information bias  (non-differential, dependent) | Blinding of outcome assessors  Prospective design and outcome assessors unaware of exposure status  Valid and reliable measurement instrument for treatment status  - |
| A common effect: treatment and outcome affect (self-)exclusion before or during the study | Attrition bias  Incomplete outcome data  Missing outcome data  Performance bias* | No exclusion of eligible patients after run-in period/ Complete follow-up > complete outcome data/Intention-to-treat analysis  Blinding of treatment of health care team | Selection bias  Survival bias | Imputation of outcome for persons that were lost to follow-up |
| Selective reporting $ | Selection of reported result | Prespecified outcomes are reported accordingly | Publication bias | No data-mining |

^ Blinding or masking is sometimes assumed to prevent allocators from foreseeing the next allocation, but this only works if the allocator is also the health professional seeing to the patient during the trial and does not know the allocation sequence; * Protocol deviations lead to differences between the allocated and actual treatment; $ cannot be depicted in a causal diagram

**eFigure 1 Bias due to not recruiting eligible patients based on knowledge of their characteristics and the next allocated intended intervention**

**?**

**O**

**I**

**C**

**S**

C stands for common cause (patient characteristic), I for **intended** intervention status, O for outcome status, and S for the common effect (de-)selection. The arrow with question mark stands for the causal question (effect) of interest. The box around S signifies that exclusion of patients occurred based on C and (knowledge of) the next allocated intended intervention. As a result, C is associated with I through the open back-door path from I to S to C. The bias can be avoided by adequate concealment of the allocation list. If (de-)selection occurred, the bias can be minimized by adjusting for (well-measured) C. In this situation, S is not a common effect of the intervention I and outcome O.
